# Supplementary material for: How we see electronic games
Source: PeerJ. 2016 Apr 11;4:e1931. doi: 10.7717/peerj.1931 (PMC4830233; doi:10.7717/peerj.1931)
Supplement: Supplemental Information 6 [file peerj-04-1931-s006.docx]

**Appendix A**

1. [They] can be a cause of real-world violence and aggression.
2. [They] can be an useful outlet for frustrations and aggression.
3. [They] are a waste of time.
4. [They] are a good form of entertainment.
5. [They] damage and reduce morals.
6. [They] negatively impact on productivity.
7. [They] promote friendship.
8. [They] develop creativity, and thinking skills.

*Note*. Principle components analysis indicated these eight items composed two attitudinal factors: 1,3,5,and 6 loaded on a factor reflecting negative gaming attitudes and items 2,4,7, and 8 loaded onto a factor reflecting positive attitudes regarding gaming.

**Appendix B**

1. [They] can be a cause of real-world violence and aggression.*
2. [They] can be a useful outlet for frustrations and aggression.
3. [They] are a waste of time.*
4. [They] are a good form of entertainment.

*Note*: Principle components analysis indicated these four items loaded onto one attitudinal factor. Items marked with a (*) were reverse coded when creating a composite reflecting positive attitudes regarding gaming.
